# Differentiation of Plant Cells During Symbiotic Nitrogen Fixation

**DOI:** 10.1002/cfg.155

**Published:** 2002-04

**Authors:** Ben Trevaskis, Gillian Colebatch, Guilhem Desbrosses, Maren Wandrey, Stefanie Wienkoop, Gerhard Saalbach, Michael Udvardi

**Affiliations:** 1 Max Planck Institute of Molecular Plant Physiology Am Mühlenberg 1 Golm 14476 Germany; 2 Risoe National Laboratory Plant biology and Biogeochemistry Department Roskilde DK-4000 Denmark

## Abstract

Nitrogen-fixing symbioses between legumes and bacteria of the family Rhizobiaceae involve differentiation of both plant and bacterial cells. Differentiation of plant root cells is required to build an organ, the nodule, which can feed and accommodate a large population of bacteria under conditions conducive to nitrogen fixation. An efficient vascular system is built to connect the nodule to the root, which delivers sugars and other nutrients to the nodule and removes the products of nitrogen fixation for use in the rest of the plant. Cells in the outer cortex differentiate to form a barrier to oxygen diffusion into nodules, which helps to produce the micro-aerobic environment necessary for bacterial nitrogenase activity. Cells of the central, infected zone of nodules undergo multiple rounds of endoreduplication, which may be necessary for colonisation by rhizobia and may enable enlargement and greater metabolic activity of these cells. Infected cells of the nodule contain rhizobia within a unique plant membrane called the peribacteroid or symbiosome membrane, which separates the bacteria from the host cell cytoplasm and mediates nutrient and signal exchanges between the partners. Rhizobia also undergo differentiation during nodule development. Not surprisingly, perhaps, differentiation of each partner is dependent upon interactions with the other. High-throughput methods to assay gene transcripts, proteins, and metabolites are now being used to explore further the different aspects of plant and bacterial differentiation. In this review, we highlight recent advances in our understanding of plant cell differentiation during nodulation that have been made, at least in part, using high-throughput methods.


**Comparative and Functional Genomics**
*Comp Funct Genom* 2002; **3**: 151–157.
Published online 12 March 2002 in Wiley InterScience (www.interscience.wiley.com). **DOI:** 10.1002/cfg.155

**Conference Review**

### Differentiation of plant cells during symbiotic nitrogen fixation

Ben Trevaskis[1], Gillian Colebatch[1], Guilhem Desbrosses[1], Maren Wandrey[1], Stefanie Wienkoop[2], Gerhard Saalbach[2] and Michael Udvardi[1]*

[1] *Max Planck Institute of Molecular Plant Physiology, Am Mühlenberg 1, 14476 Golm, Germany*
[2] *Risoe National Laboratory, Plant biology and Biogeochemistry Department, DK-4000 Roskilde, Denmark*

**Correspondence to:*
*Max Planck Institute of Molecular Plant Physiology, Am Mühlenberg 1, 14476 Golm, Germany.*
*E-mail:*
*Udvardi@mpimp-golm.mpg.de*

Received: 11 February 2002
Accepted: 12 February 2002

### Abstract

**Nitrogen-fixing symbioses between legumes and bacteria of the family Rhizobiaceae involve differentiation of both plant and bacterial cells. Differentiation of plant root cells is required to build an organ, the nodule, which can feed and accommodate a large population of bacteria under conditions conducive to nitrogen fixation. An efficient vascular system is built to connect the nodule to the root, which delivers sugars and other nutrients to the nodule and removes the products of nitrogen fixation for use in the rest of the plant. Cells in the outer cortex differentiate to form a barrier to oxygen diffusion into nodules, which helps to produce the micro-aerobic environment necessary for bacterial nitrogenase activity. Cells of the central, infected zone of nodules undergo multiple rounds of endoreduplication, which may be necessary for colonisation by rhizobia and may enable enlargement and greater metabolic activity of these cells. Infected cells of the nodule contain rhizobia within a unique plant membrane called the peribacteroid or symbiosome membrane, which separates the bacteria from the host cell cytoplasm and mediates nutrient and signal exchanges between the partners. Rhizobia also undergo differentiation during nodule development. Not surprisingly, perhaps, differentiation of each partner is dependent upon interactions with the other. High-throughput methods to assay gene transcripts, proteins, and metabolites are now being used to explore further the different aspects of plant and bacterial differentiation. In this review, we highlight recent advances in our understanding of plant cell differentiation during nodulation that have been made, at least in part, using high-throughput methods.** **Copyright © 2002 John Wiley & Sons, Ltd.**

**Keywords:** **symbiosis; nitrogen-fixation; differentiation; transcriptomics; metabolomics; proteomics**

### Introduction

Nitrogen is an essential element for plant growth and reproduction. Lack of mineral nitrogen in the soil often limits plant growth and, as a result, modern agriculture uses approximately 80 million tons of nitrogen fertilizer annually to maintain the world's food supply. However, some plants are able to utilize the enormous pool of gaseous $N_2$ present in the atmosphere by forming symbioses with nitrogen-fixing bacteria, which produce ammonium for the plant. Such plants thrive in nitrogen-poor soils and contribute significantly to the global nitrogen cycle and to agriculture. The best-known nitrogen-fixing symbioses occur between legumes (*Fabaceae*) and bacteria of the *Rhizobiaceae* family.

The legume-rhizobia symbiosis is initiated by the exchange of signal molecules between the plant and microbe. Flavonoid compounds released by the plant attract rhizobia and trigger the production and release of nodulation (Nod) factors by the bacteria [42]. Nod factors are specific lipochito-oligosacharrides [32] that affect the host plant in a number of ways. The first visible effect of Nod factors on the plant root is the curling of root hairs. Nod factors also trigger cell division in cortical cells, which leads to the formation of the nodule meristem [9,13,46].

Copyright © 2002 John Wiley & Sons, Ltd.

Rhizobia bound to curled root hairs cause localised cell wall degradation and enter the plant root via a specialised extracellular structure known as the infection thread, which grows through the root towards dividing cells, the nodule primordia, within the root cortex [4]. When the rhizobia reach the nodule promordia they infect root cells and are internalised within the peribacteroid or symbiosome membrane [4,11]. Within these organelle-like compartments the bacteria divide and differentiate into nitrogen-fixing bacteroids [55]. Cell division continues in the root tissue also, eventually forming an outgrowth containing the infected plant cells. The infected cells are surrounded by uninfected tissue that contains vascular tissue for the delivery and export of metabolites to the nodule. Another uninfected cell layer forms an oxygen barrier that helps to protect the oxygen sensitive nitrogenase enzyme [29,40]. Within the infected cells of a mature nodule the bacteroids fix nitrogen in return for nutrients from the plant [52].

There are differences between the nodule developmental programmes of different legumes. In the case of indeterminate nodules, such as the nodules formed by pea or *Medicago truncatula*, the nodule meristem differentiates from inner-cortical cells and is persistent, with new cells being constantly produced and infected. In determinate nodules, such as those of soybean or *Lotus japonicus*, the nodule meristem differentiates from cells in the outer cortex and is only transiently active. The infected region of a determinate nodule contains infected host cells that are all at a similar developmental stage [3,23].

Nodule meristems produce two major types of tissue: the central tissue containing infected and uninfected cells; and the peripheral tissue consisting of cortical, endodermal, and parenchymal cells, which contain the vascular bundles [37]. The central tissue of indeterminate nodules can be divided into several zones, differing in their degree of cell differentiation: the meristematic zone; the pre-fixation or invasion zone, where cell division is arrested and cells differentiate and are invaded; the nitrogen fixation zone, where plant cells and bacteria are terminally differentiated for nitrogen fixation; and the senescent zone, closest to the root, which contains degenerated plant and bacterial cells [55]. Cells of the meristem contain 2C-4C DNA, depending upon whether they are in G1 or G2, whilst cells in the other zones are endoreduplicated [6]. Increased ploidy levels may promote infection or allow increased cell size and metabolic activity in infected cells [6]. In contrast to the situation in indeterminate nodules, cells of the infected zone of determinate nodules differentiate in unison. The infected zone of determinate nodules also contains polyploid cells [3].

The study of rhizobia has yielded a wealth of information. The entire genomes of *Sinorhizobium meliloti*, the symbiotic partner of Medicago, and of *Mezorhizobium loti*, the symbiotic partner of Lotus, have been sequenced [19,28]. The roles and regulation of many genes required for effective symbiosis are well understood. Genes required for nodulation (*nod* genes) [34,12], metabolism of dicarboxylic acids (*dct* genes) [57,58], and synthesis or activity of the nitrogenase complex (*nif* and *fix* genes) [26], are examples of rhizobial genes that have been found to be essential for symbiosis. Many of these genes are induced in the differentiating rhizobia during symbiosis. Progress in understanding plant-controlled aspects of nodule development has been slower, hindered by longer generation times and by the added complexity of the eukaryotic system. It is clear that the plant host controls many aspects of nodule development and in some legumes nodule-like structures can spontaneously develop in the complete absence of rhizobia [51]. Recent advances in plant genomics are starting to accelerate progress in understanding plant-controlled aspects of the legume-rhizobia nitrogen fixing symbiosis. In this review we highlight past work in this area and describe more recent results obtained with high-throughput molecular methods that provide new insights into plant cell differentiation during symbiotic nitrogen fixation.

### Nodulins

The term nodulin was originally used to describe 'nodule-specific host polypeptides' [31]. Genes encoding nodule-specific proteins are likely to play important roles in nodule differentiation and development. Nodulin genes have been isolated using a number of methods of molecular biology, including cold-plaque screening [16,27], mRNA differential display [48], subtractive hybridisation [20,30] and differential screening of nodule cDNA [18,41]. Nodulin genes have been classified into two groups: those that are induced soon after contact with rhizobia, the early nodulins: and those expressed late during the nodulation process or in mature nodules, the late nodulins. Early nodulins are likely to be involved in

Copyright © 2002 John Wiley & Sons, Ltd. 

the early stages of nodule development, such as cortical cell division and infection thread synthesis, and include several proline-rich structural proteins of the cell wall [33,45]. The classic late nodulin is leghemoglobin, which is found in high concentrations in the cytoplasm of infected cells of mature nodules where it binds oxygen and buffers free oxygen levels to prevent inactivation of the oxygen sensitive nitrogenase enzyme [1]. Other late nodulins appear to be involved in primary nitrogen and carbon metabolism, as well as transport of nutrients between the bacteria and plant [54,25,49]. It is now clear that only a few of the genes that are up-regulated during nodule development are expressed exclusively in nodules. Many previously described nodulins have been shown to be expressed in other plant tissues when assayed with more sensitive expression analysis techniques such as RT-PCR (e.g. [50]). In this respect the original nodulin concept is over-simplistic; in reality there are probably few genes that are expressed exclusively in nodules. Regardless of whether these genes are nodule specific or up-regulated in nodules, they are likely to be important for symbiotic nitrogen fixation. Nodulin genes have also been useful as markers for examining various aspects of the early nodulation process, such as the potential involvement of plant hormones [35] and for determining which processes are affected in mutants that are defective in the nodule differentiation process, the analysis of the DMI mutants for example [5].

### Transcriptome analysis

The genes that have previously been identified as nodulins represent only a subset of all of the genes that are induced during nodule differentiation and development. High-throughput methods for expression analysis are now being used to compare gene expression between symbiotic and non-symbiotic plant tissues, and thus to identify large numbers of genes that are up-regulated during nodule development. One such method relies on DNA arrays, on which thousands of genes or gene fragments are placed and used as ‘probes’ to detect the amount of specific transcripts in a given RNA sample. For example, we have produced nylon membranes containing 2300 *Lotus japonicus* nodule cDNAs, and have used them to identify 83 genes that were up-regulated in mature nodules compared to roots [10]. These included several nodulin genes of unknown function. Also included were genes involved in sucrose breakdown and glycolysis, $CO_2$ recycling, and amino acid synthesis, which are all processes that are accelerated in nodules compared to roots. Genes involved in membrane transport, hormone metabolism, cell wall and protein synthesis, signal transduction, and regulation of transcription were also induced in nodules. Genes that may subvert normal plant defence responses, including two encoding enzymes involved in detoxification of active oxygen intermediates, and one that may prohibit phytoalexin synthesis were also identified. More than half of the genes identified in our work have never before been identified as induced in nodules. Lotus transcript profiling also uncovered genes that were repressed during nodule development and differentiation. In fact, many of the genes examined were expressed at higher levels in roots than nodules (unpublished). This may reflect the specialised function of nodules in nitrogen acquisition. Thus, DNA array analysis is providing multiple new leads for symbiosis research.

### Proteomics and the symbiotic interface

A key difference between infected cells of legume root nodules and all other plant cell types is the presence in the former of nitrogen-fixing ‘organelles’ called symbiosomes, which consist of one or more bacteroids surrounded by the plant-derived symbiosome membrane [11]. The symbiosome membrane is the interface between plant and bacteroids, and is likely to contain many proteins essential for nutrient and signal exchange between the plant and bacteroids [52]. Several previously identified nodulin proteins are located in and on the symbiosome membrane, including Nodulin 26, an aquaglyceroporin protein whose exact function is unclear [15,21]. There are likely to be many more as yet unidentified proteins on this membrane that fulfill functions critical to symbiotic nitrogen fixation.

Systematic approaches are now available to identify proteins in biological samples. The most effective of these, in terms of throughput and sensitivity, are based on mass spectrometry (MS), which can determine the masses of protein fragments produced by protease digestion or the sequence of small peptides, both of which can serve as a ‘fingerprint’ to identify the protein of interest [38,59]. In many instances the identification of

Copyright © 2002 John Wiley & Sons, Ltd. 

proteins has been aided greatly by access to genome sequence of the organism being studied. For organisms such as yeast, whose genomes have been completely sequenced, identification of thousands of different proteins can be done quickly and routinely [56] and quantification of proteins is also possible [22]. This new field of work has been dubbed 'proteomics'.

We are using proteomics to study proteins on the symbiosome membrane. Utilizing methods to purify symbiosome membranes from the legumes soybean and *Lotus japonicus* [53,8], together with proteomics tools, we have identified numerous proteins associated with the symbiosome membrane. Some of these are peripheral membrane proteins [39], while others are integral membrane proteins, including Nodulin 26 as well as a putative cation channel regulator from soybean and a sulfate transporter homologue from Lotus (unpublished). Interestingly, the putative sulfate transporter matched precisely the protein encoded by one of the genes that we identified as nodule-induced using Lotus cDNA arrays. Real-time RT-PCR analysis showed that transcripts from this gene were 500-fold higher in mature nodules than in uninfected roots [10]. This type of result may turn out to be common for genes encoding symbiosome membrane proteins because of the highly specialised role of the membrane and the proteins it contains. Our results provide a nice example of the way in which transcriptome and proteome analysis can complement one another in the quest to document and understand cellular differentiation. Another complementary analytical tool is provided by the discipline of metabolomics, described briefly below.

### Metabolomics and nodule cell differentiation

Unlike roots, which acquire a range of essential mineral nutrients from the soil, nodules are specialised in nitrogen acquisition and are produced only when mineral nitrogen is limiting. Such specialisation might be expected to be reflected by a more streamlined metabolism, carried out by fewer proteins and orchestrated by fewer genes. Over the past few years, the tools of analytical chemistry have been developed to the point that high-throughput analysis of hundreds to thousands of metabolites is now possible [14]. Such analyses typically employ either liquid or gas chromatography in series with mass spectrometry [14,43]. We have used GC-MS to profile metabolites present in nodules and other organs of Lotus. Each organ exhibits a different metabolite profile, although, of course, the profile changes with organ development and physiological status. Detailed analysis of nodule and root metabolite profiles may help us to identify novel aspects of nodule metabolism that underpin symbiotic nitrogen fixation. For example, our comparison of root and nodule transcript levels in Lotus has uncovered nodule-induced genes encoding enzymes that have not previously been associated with symbiotic nitrogen fixation. Quantification of the reactants and products of these enzymes may provide some insight into their importance in nodules. Metabolomics is also likely to prove useful in analysing the nodule phenotypes of symbiotic interactions formed with either mutant bacteria or mutant plants. In yeast it has been shown that seemingly silent mutations show clear phenotypes at the metabolome level [43].

### Classical and reverse genetics

Classical genetic analysis of legumes has been successful in identifying loci that are either essential for nodule development or for effective nitrogen fixation. Hypernodulating mutants have also been identified [47]. By examining mutants for defects in visible features of nodule development, such as root hair curling or cortical cell division, and examining expression of early nodulin genes, it has been possible in many instances to determine where in the nodulation process such mutants are blocked. The first plant gene essential for nitrogen fixing symbiosis has now been isolated, the *Nin* gene of Lotus, which encodes a transcription factor that is essential for initiating cortical cell division [44]. The *Nin* gene was cloned using a transposon tag, and with more tagged lines being generated in Lotus it is likely that mutants defective in other loci essential for symbiotic nitrogen fixation will be identified soon. Increasingly detailed genetic maps in model legume species such as Lotus and Medicago, combined with increased genome and EST sequencing efforts are also facilitating map based cloning and it is likely that a number of interesting genes involved in nodulation will be cloned in the near future [47].

Transformation protocols have been developed for a number of legume species, including *Lotus*

Copyright © 2002 John Wiley & Sons, Ltd. 

*japonicus* and *Medicago truncatula*, which enable reverse genetics to be used to study nodule development [24,2]. Obvious targets for reverse genetics are nodulin or nodule-induced genes, several of which have now been studied. The early nodulin gene *enod40* is induced by Nod factors and expressed in nodule primordia in pea [36]. Constitutive over-expression of *enod40* induced dedifferentiation and division of cortical cells in *Medicago truncatula*, suggesting a role for the protein in nodule initiation [7]. Anti-sense suppression of the transcription factor *Mszpt2-1*, which is normally expressed in vascular bundles of developing alfalfa nodules, resulted in non-functional nodules in which differentiation of the nitrogen fixing zone and bacterial invasion were arrested [17]. The *ccs52* gene, which encodes an inhibitor of mitosis, was also implicated in cellular differentiation in legume nodules by reverse genetics. Expression of *ccs52* was enhanced in cells undergoing endoreduplication. Anti-sense suppression of the gene in Medicago resulted in a decreased number of endocycles and reduced volume of the largest nodule cells [6]. Thus, *ccs52* appears to be necessary for endoreduplication and differentiation of nodule cells.

### Outlook

The high-throughput technologies of functional genomics are opening our eyes to the true complexity of cellular differentiation that underpins symbiotic nitrogen fixation. Integration of results of transcriptome, proteome, and metabolome analyses with those obtained from forward and reverse genetics will undoubtedly lead to a better understanding of nitrogen-fixing symbioses. Ultimately, this may lead to an extension of symbiotic fixation of nitrogen to agriculturally important non-legumes.

### Acknowledgement

The Max Planck Society, Deutsche Forschungsgemeinschaft, Alexander von Humboldt Society, and European Union are thanked for their support of this work.

### References

1. Appleby O. 1984. Leghemoglobin and *Rhizobium* respiration. *Annu Rev plant Phys* **35**: 443–478.
2. Barker DG, Bianchi S, Blondon F, *et al*. 1990. *Medicago truncatula*, a model plant for studying the molecular genetics of the *Rhizobium*-legume symbiosis. *Plant Mol Bio Rep* **8**: 40–49.
3. Brewin NJ. 1991. Development of the legume root nodule. *Ann Rev Cell Biol* **7**: 191–226.
4. Brewin NJ. 1998. Tissue and cell invasion by *Rhizobium*: the structure and development of infection threads and symbiosomes. In *The Rhizobiacea, the molecular biology of model plant associated bacteria*, Spaink HP, Kondorosi A, Hooykaas PJJ (eds). Kluver Academic Publishers: Dordretch; 417–429.
5. Catoira R, Galera C, de Billy F, *et al*. 2000. Four genes of *Medicago truncatula* controlling components of a nod factor transduction pathway. *Plant Cell* **12**: 1647–1666.
6. Cebolla A, Vinardell JM, Kiss E, *et al*. 1999. The mitotic inhibitor *ccs52* is required for endoreduplication and ploidy-dependent cell enlargement in plants. *Embo J* **18**: 4476–4484.
7. Charon C, Johansson C, Kondorosi E, Kondorosi A, Crespi M. 1997. *enod40* induces dedifferentiation and division of root cortical cells in legumes. *Proc Natl Acad Sci U S A* **94**: 8901–8906.
8. Christiansen JH, Rosendahl L, Widell S. 1995. Preparation and characterization of sealed inside-out peribacteroid membrane vesicles from *Pisum sativum L.* and *Glycine max L.* root nodules by aqueous polymer two-phase partitioning. *Plant Physiol* **147**: 175–181.
9. Cohn J, Day RB, Stacey G. 1998. Legume nodule organogenesis. *Trends in Plant Science* **3**: 105–110.
10. Colebatch G, Kloska S, Trevaskis B, *et al*. 2002. Novel aspects of symbiotic nitrogen fixation uncovered by transcript profiling with cDNA arrays. *Mol plant Microbe Interact* (in press).
11. Day DA, Price GD, Udvardi MK. 1989. Membrane interface of the *Brazyrhizobium japonicum-Glycine max* symbiosis: peribacteroid membrane units from soybean nodules. *Aust J Plant Physiol* **16**: 69–84.
12. Debelle F, Moulin L, Mangin B, Denarie J, Boivin C. 2001. Nod genes and Nod signals and the evolution of the *Rhizobium* legume symbiosis. *Acta Biochim Pol* **48**: 359–365.
13. Denarie J, Debelle F, Prome JC. 1996. *Rhizobium* lipo-chitooligosaccharide nodulation factors: signaling molecules mediating recognition and morphogenesis. *Annu Rev Biochem* **65**: 503–535.
14. Fiehn O, Kopka J, Dormann P, *et al*. 2000. Metabolite profiling for plant functional genomics. *Nat Biotechnol* **18**: 1157–1161.
15. Fortin MG, Morrison NA, Verma DP. 1987. Nodulin-26, a peribacteroid membrane nodulin is expressed independently of the development of the peribacteroid compartment. *Nucleic Acids Res* **15**: 813–824.
16. Frugier F, Kondorosi A, Crespi M. 1998. Identification of novel putative regulatory genes induced during alfalfa nodule development with a cold-plaque screening procedure. *Mol Plant Microbe Interact* **11**: 358–366.
17. Frugier F, Poirier S, Satiat-Jeunemaitre B, Kondorosi A, Crespi M. 2000. A Kruppel-like zinc finger protein is involved in nitrogen-fixing root nodule organogenesis. *Genes Dev* **14**: 475–482.
18. Fuller F, Kuenstner PW, Nguyen T, Verma DPS. 1983. Soybean nodulin genes: Analysis of cDNA clones reveals several major tissue-specific sequences in nitrogen-fixing root nodules. *Proc Natl Acad Sci U S A* **80**: 2594.

Copyright © 2002 John Wiley & Sons, Ltd. 

19. Galibert F, Finan TM, Long SR, *et al.* 2001. The composite genome of the legume symbiont *Sinorhizobium meliloti*. *Science* **293**: 668–672.
20. Gamas P, Niebel F, Lescure N, Cullimore J. 1996. Use of a subtractive hybridization approach to identify new *Medicago truncatula* genes induced during root nodule development. *Mol Plant Microbe Interact* **9**: 233–242.
21. Guenther JF, Roberts DM. 2000. Water-selective and multi-functional aquaporins from *Lotus japonicus* nodules. *Planta* **210**: 741–748.
22. Gygi SP, Rist B, Gerber SA, *et al.* 1999. Quantitative analysis of complex protein mixtures using isotope-coded affinity tags. *Nat Biotechnol* **17**: 994–999.
23. Hadri A, Spaink H, Bisseling T, Brewin NJ. 1998. Diversity of root nodulation and rhizobial infection processes. In *The Rhizobiacea, the molecular biology of model plant associated bacteria*, Spaink HP, Kondoros A, Hooykaas PJJ (eds). Kluver Academic Publishers: Dordretch; 347–360.
24. Handberg K, Stiller J, Thykjear T, Stougaard J. 1994. Transgenic plants, *Agrobacterium* mediated transformation of the diploid legume *Lotus japonicus*. In *Cell Biology, a labratory handbook*, Cellis JE (ed.). Academic Press: New York; 119–150.
25. Hata S, Izui K, Kouchi H. 1998. Expression of a soybean nodule-enhanced phosphoenolpyruvate carboxylase gene that shows striking similarity to another gene for a house-keeping isoform. *Plant J* **13**: 267–273.
26. Hirsch AM, Smith CA. 1987. Effects of *Rhizobium meliloti* nif and fix mutants on alfalfa root nodule development. *J Bacteriol* **169**: 1137–1146.
27. Jimenez-Zurdo JI, Frugier F, Crespi MD, Kondorosi A. 2000. Expression profiles of 22 novel molecular markers for organogenetic pathways acting in alfalfa nodule development. *Mol Plant Microbe Interact* **13**: 96–106.
28. Kaneko T, Nakamura Y, Sato S, *et al.* 2000. Complete genome structure of the nitrogen-fixing symbiotic bacterium *Mesorhizobium loti*. *DNA Research* **7**: 331–338.
29. King BJ, Hunt S, Waegle GE, *et al.* 1988. Regulation of $O_2$ concentration of soybean nodules observed by *In situ* spectroscopic measurements of leghemoglobin oxygenation. *Plant Physiol* **87**: 296–299.
30. Kouchi H, Hata S. 1993. Isolation and characterization of novel nodulin cDNAs representing genes expressed at early stages of soybean nodule development. *Mol Gen Genet* **238**: 106–119.
31. Legocki RP, Verma DP. 1980. Identification of 'nodule-specific' host proteins (nodulins) involved in the development of *Rhizobium*-legume symbiosis. *Cell* **20**: 153–163.
32. Lerouge P, Roche P, Faucher C, *et al.* 1990. Symbiotic host-specificity of *Rhizobium meliloti* is determined by a sulphated and acylated glucosamine oligosaccharide signal. *Nature* **344**: 781–784.
33. Löbler M, Hirsch AM. 1993. A gene that encodes a proline-rich nodulin with limited homology to PsENOD12 is expressed in the invasion zone of *Rhizobium meliloti*-induced alfalfa root nodules. *Plant Physiol* **103**: 21–30.
34. Long SR. 1996. *Rhizobium* Symbiosis: Nod factors in perspective. *The Plant Cell* **8**: 1885–1898.
35. Mathesius U, Charon C, Rolfe BG, Kondorosi A, Crespi M. 2000. Temporal and spatial order of events during the induction of cortical cell divisions in white clover by *Rhizobium leguminosarum bv. trifolii* inoculation or localized cytokinin addition. *Mol Plant Microbe Interact* **13**: 617–628.
36. Matvienko M, Van de Sande K, Yang WC, *et al.* 1994. Comparison of soybean and pea *ENOD40* cDNA clones representing genes expressed during both early and late stages of nodule development. *Plant Mol Biol* **26**: 487–493.
37. Newcomb W, Mclntyre L. 1981. Development of root nodules of mung bean (*Vigan radiata*): a reinvestigation of endocytosis. *Canadian Journal of Botany* **59**: 2478–2499.
38. Pandey A, Mann M. 2000. Proteomics to study genes and genomes. *Nature* **405**: 837–846.
39. Panter S, Thomson R, de Bruxelles G, *et al.* 2000. Identification with proteomics of novel proteins associated with the peribacteroid membrane of soybean root nodules. *Mol Plant Microbe Interact* **13**: 325–333.
40. Parsons R, Day DA. 1990. Mechanism of soybean nodule adaptation to different oxygen pressure. *Plant Cell and Environment* **13**: 501–512.
41. Perlick AM, Puhler A. 1993. A survey of transcripts expressed specifically in root nodules of broadbean (*Vicia faba* L.). *Plant Mol Biol* **22**: 957–970.
42. Peters NK, Frost JW, Long SR. 1986. A plant flavone, luteolin, induces expression of *Rhizobium meliloti* nodulation genes. *Science* **233**: 977–980.
43. Raamsdonk LM, Teusink B, Broadhurst D, *et al.* 2001. A functional genomics strategy that uses metabolome data to reveal the phenotype of silent mutations. *Nat Biotechnol* **19**: 45–50.
44. Schauser L, Roussis A, Stiller J, Stougaard J. 1999. A plant regulator controlling development of symbiotic root nodules. *Nature* **402**: 191–195.
45. Scheres B, van Engelen F, van der Knaap E, *et al.* 1990. Sequential induction of nodulin gene expression in the developing pea nodule. *Plant Cell* **2**: 687–700.
46. Schultze M, Kondorosi A. 1998. Regulation of symbiotic root nodule development. *Annu Rev Genet* **32**: 33–57.
47. Stougaard J. 2001. Genetics and genomics of root symbiosis. *Curr Opin Plant Biol* **4**: 328–335.
48. Szczyglowski K, Hamburger D, Kapranov P, de Bruijn FJ. 1997. Construction of a *Lotus japonicus* late nodulin expressed sequence tag library and identification of novel nodule-specific genes. *Plant Physiol* **114**: 1335–1346.
49. Szczyglowski K, Kapranov P, Hamburger D, de Bruijn FJ. 1998. The *Lotus japonicus LjNOD70* nodulin gene encodes a protein with similarities to transporters. *Plant Mol Biol* **37**: 651–661.
50. Takane K, Tanaka K, Tajima S, Okazaki K, Kouchi H. 1997. Expression of a gene for uricase II (nodulin-35) in cotyledons of soybean plants. *Plant Cell Physiol* **38**: 149–154.
51. Truchet G, Camut S, de Billy F, Odorico R, Vasse J. 1989. Alfalfa nodulation in the abscence of *Rhizobium*. *Mol Gen Genet* **219**: 65–68.
52. Udvardi MK, Day DA. 1997. Metabolite transport across symbiotic membranes of legume nodules. *Annu Rev of Plant Physiol Plant Molecular* **00**:493–523.
53. Udvardi MK, Price D, Gresshoff PM, Day DA. 1988. A dicarboxylate transporter on the peribacteroid membrane of soybean nodules. *FEBS Lett.* **231**: 36–40.
54. Vance CP, Gregerson RG, Robinson DL, Miller SS, Gantt JS. 1994. Primary assimilation of nitrogen in alfalfa nodules:

Copyright © 2002 John Wiley & Sons, Ltd.

molecular features of the enzymes involved. *Plant Science* **101**: 51–64.

55. Vasse J, de Billy F, Camut S, Truchet G. 1990. Correlation between ultrastructural differentiation of bacteroids and nitrogen fixation in alfalfa nodules. *J Bacteriol* **172**: 4295–4306.
56. Washburn MP, Wolters D, Yates JR. 2001. Large-scale analysis of the yeast proteome by multidimensional protein identification technology. *Nat Biotechnol* **19**: 242–247.
57. Watson RJ, Chan YK, Wheatcroft R, Yang AF, Han SH. 1988. *Rhizobium* meliloti genes required for C4-dicarboxylate transport and symbiotic nitrogen fixation are located on a megaplasmid. *J Bacteriol* **170**: 927–934.
58. Yarosh OK, Charles TC, Finan TM. 1989. Analysis of C4-dicarboxylate transport genes in *Rhizobium meliloti*. *Mol-Microbiol* **3**: 813–823.
59. Yates JR. 2000. Mass spectrometry. From genomics to proteomics. *Trends Genet* **16**: 5–8.

Copyright © 2002 John Wiley & Sons, Ltd. 
